# Observations on the Structure and Diseases of the Testis

**Published:** 1830-10-01

**Authors:** 


**Observations on the Structure and Diseases of the Testis. By *Sir Astley Cooper*, Bart. F.R.S. Serjeant-Surgeon to the King, Consulting Surgeon to Guy's Hospital, &c. &c. Quarto, pp. 245; 22 Plates. Longman's, London, 1830.**

The medical reviewer has oftentimes a pleasing, but oftener an unpalatable task. The number of good books which issue from the press bear a wofully small proportion to the bad and the indifferent; but the honest critic must wade through all, and expend his time and labour upon many. This is mere drudgery, and of drudgery we do not complain, for it is our business to undergo it. But it is a curious and by no means inexplicable fact, that the worse the book the more importunate and exorbitant in his expectations is the author, and many " a puddle in a storm" is it our fate to witness and our task to appease. An able author is altogether a different personage to deal with. He is strong and he knows it; having nothing to dread from criticism or the critic, he neither annoys the one nor is galled and nettled by the other. In short, as a real gentleman invariably gives less trouble than a would-be one, so the author of a valuable work is infinitely more tractable than the writer of a volume of trash. We believe we have contrived to give as little offence as any of our editorial confréres; but God knows we stand upon ticklish ground, and, like the Roman legions in the Caudine forks, a single faux-pas sends us under the yoke of an irritable author's ire.

These reflections were naturally excited by the contemplation of the magnificent volume before us. In a preface, displaying the modesty which real merit always wears, Sir Astley Cooper observes, that they who are aware of the expenses of such works, require not to be told that his object in publishing cannot be pecuniary advantage. Having been placed for forty years in a situation of ample opportunity—having been fostered by the profession and the public infinitely beyond his deserts—he feels that he is only performing his duty in giving to his medical brethren, without any sordid views, the result of his experience. How plain, and manly, and noble a sentiment! How honourable to the veteran who has fought, we may say, the battles of science for nearly half a century, to dedicate his trophies to the Commonwealth, and expend his latter years in bestowing information and conferring benefits on those who shall come after him. None can fail to be struck by the contrast between such a man and the fry of small authors, whose flippant étourderies too frequently disgrace the medical periodical press. A contributor of a false fact to the most obscure journal, would make more pother and raise more dust with his stupid egotism, than all the distinguished authors of ancient or modern times put together.

The present work on the diseases of the testis is divided into two parts. The first is occupied by a copious, and in many respects novel, description of the anatomy of the healthy organ—in the second, its diseases are treated of at large. We intend to give a full, yet, we trust, succinct account of *all* the contents of this splendid volume, but we cannot pursue the order of the able author. We shall, therefore, commence with the diseases of the testis, and conclude with its anatomy, by which means we shall place the practical details in the hands of our remote and less affluent brethren, at an earlier period than if we adopted a different arrangement. Perhaps even this slight alteration may prove the means of benefit to individuals affected with these maladies, nay, spare them the horrors and the dangers of an unnecessary operation.

In looking at some classes of diseases, the present generation have much reason to be proud of the advances which zeal, industry, observation, and talent have effected. We might instance many, but shall here content ourselves with those more immediately under consideration. It is not twenty, no, nor ten years since, that the chronic inflammation of the testicle was looked upon with awe as a "sarcocele," and many a testicle was extirpated then, which is now preserved by the timely and judicious exhibition of mercury. Surely nothing can afford more pleasure to an ingenuous and humane mind, than the thought that numbers of individuals are no longer submitted to pangs and maimings which were daily inflicted by our ancestors. In a pathological point of view, we have witnessed equal improvement in the discrimination of the various organic changes to which the testicle is subject; and, although it may not be now of such service to distinguish scirrhus of the gland from fungus hæmatodes, yet accuracy of diagnosis is never attended with any evils, and may be followed by important consequences. Of the labourers in this vineyard, we must rank Sir Astley Cooper, not among the foremost, but the very first. His former publication, on the diseases of the breast, and his present volume, will probably confer more benefit on practical surgery than any contemporary writings. This is no compliment, it is truth from the mouths of those who have nothing to gain by flattery, nothing to lose by disapprobation.

In the illustrations of the complaints of the breast before referred to, Sir Astley treated only of the non-malignant. He has delayed the second part, containing an account of the malignant, because there are some points on which he is not yet fully satisfied. The present observations on the testicle will be found in part in his published lectures,* but much has been added, and the plates are, of course, altogether new. The morbid preparations, illustrating the able Baronet's descriptions, are placed in the museum of St. Thomas's Hospital, and in excellent preservation they remain. With these observations and explanations we proceed to work, we mean to analysis. No pains shall be spared to render this as close as possible, and place the substance of a three-guinea volume before the readers of this Journal. This diffusion of his opinions will give more pleasure to Sir Astley Cooper, than the sordid gain which the sale of thousands of copies could afford. The first chapter consists of—

### GENERAL OBSERVATIONS ON THE DISEASES OF THE TESTIS.

Sir Astley divides the diseases of the testis, like those of the breast, first

---

* By Tyrrell. By the way, when is the fourth volume to appear? There is a wonderfully long interval between the births.

into those which result from common inflammation, acute or chronic;—secondly, into the specific, but non-malignant, the action differing from common inflammation;—thirdly, into the specific and malignant.

The acute inflammation after having spent its force retires, and leaves but little alteration in the part; it sometimes terminates by abscess which forms with rapidity, but discharges itself slowly by ulceration. The chronic inflammation is slow in the adhesive, suppurative, and ulcerative stages, and it produces sinuses and exuberant granulations, which are difficult of cure and require a peculiar management. The specific but not malignant diseases seldom proceed to suppuration. Some of them lead to the necessity of extirpation, as the hydatid disease; but others, as the mumps, the scrofulous, and perhaps the venereal disorder of the testicle yield to the action of medicine. The malignant diseases are the fungous and scirrhous, the latter, in Sir Astley's opinion, a very rare affection.

The diseases of the testicle are either local only, or "constitutionally local;" and the constitution may be also secondarily affected by the local irritation. Acute inflammation, however arising, is a local disorder, or, if the constitution be implicated, it is secondarily. Chronic inflammation originates in a peculiar state of the constitution as well as in the part, both giving a languid and tardy character to the processes of inflammation. Specific non-malignant diseases may be local only, as the hydatid or encysted; or they may be constitutional and local, as the scrofulous; but the products of both are different from those of common inflammation. In malignant diseases the disposition to them is founded on unhealthy changes in the constitution; but their chief distinction is their property of contaminating structures in contact with them, and invading contiguous parts through the medium of the absorbents; similar diseases may also exist independently in other parts. If a malignant disease be removed, the wound often heals rapidly as in a healthy person, whilst the disease reappears some time afterwards. This proves, according to Sir A., that the disease is the result, not only of constitutional predisposition, but specific local action, differing from common inflammation, which has generally subsided before the disease returns. We think that objection might be made to some items of Sir Astley's arrangement, and that some of his positions are not altogether unexceptionable. But our object is analysis not criticism, and we can reserve any remarks till we come to treat of the particular affections.

The diseases of the testicle are, 1st, common inflammation, acute or chronic; wasting of the testicle: 2dly, specific non-malignant diseases, as the hydatid or encysted; the irritable; the mumps affecting the testes; the ossific; single cysts and solid tumours of the epididymis or testes; the scrofulous; the venereal, which some may doubt: 3dly, the malignant diseases, fungoid and scirrhous. From this enumeration our author has excluded the affections of the tunics and cord, as the hydrocele, &c. which he will describe in another part of the work. The testis is less prone to disease than the breast, but the cord and tunics are subject to a great variety of complaints. Diseases of the testicle are more amenable to treatment than those of the breast, and Sir Astley allows that castration is too often unnecessarily and precipitately performed.

The causes of disease of the testicle, independent of constitutional predisposition are, first—their pendulous situation, rendering them more liable, by the gravitation of the blood, to congestion and inflammation;—secondly, the excitement of passion, often not immediately indulged, which leads to accumulation of their secretion, distention of the seminiferous tubes, and inflammation;—thirdly, exposure to mechanical injury;—fourthly, the strong natural sympathy existing between the testicles and urethra and prostate gland, rendering them very apt to inflame sympathetically: but the testis sympathises chiefly with the membranous and prostatic portions of the urethra;—fifthly, the changes which the gland undergoes at puberty and in old age, especially the latter;*—sixthly, the descent of the testicle, which is often delayed for some years after birth, and sometimes till adult age, during which time it remains in the inguinal passage or groin, exposed to injury, and prone from its unnatural situation to suffer diseased changes;—seventhly, vicissitudes of temperature. To these we will add—eighthly, hereditary predisposition; for, in a case we lately saw, the patient's grandfather and uncle by the father's side had suffered from disease of the testicle; in the patient to whom we allude, the complaint yielded to mercury.

### Acute Inflammation of the Testicle.

Hernia humoralis, the name by which this affection is usually known, is manifestly erroneous, but, as in other instances, the term is retained from habit and for convenience, when the old signification and sense are overlooked. Although the consequence of gonorrhœa, it has nothing gonorrhœal in its character, or venereal in its nature, and Sir Astley would prefer the name of testitis, if he were not afraid of appearing affected.

The first symptom of this affection, when it arises from sympathy with the urethra, is an irritation of the membranous or prostatic part of the canal, as if some drops of urine still remained in the commencement of the urethra. This is succeeded by tenderness in the cord at the ring, and by swelling and pain in the epididymis. The testis next swells to two or three times its natural bulk, becomes extremely tender, and by its weight drags painfully on the spermatic cord. The pain is obtuse, heavy, and aching, resembling that produced by squeezing the testicle, and arising from the same cause—the distention of the glandular structure, whilst the tendinous and inelastic tunica albuginea does not yield to the swelling from within. The pain and swelling extend along the cord to the inguinal canal, producing great uneasiness in the groin and spinous process of the ilium, hip, and inner part of the thigh on the affected side, and fixing itself at length more particularly in the loin. The nervous connexions will readily explain this, as well as the nausea and sometimes severe vomiting, the colicky pains in the intestines and constipation. The pain and inflammation extend to the neck of the bladder, occasioning dysuria, and frequent desire to make water. The testicle when much swollen retains its original form, but is flatter at the sides and feels excessively hard. The epididymis swells more in proportion, and continues swollen longer than the testis, which is owing to its covering being less compact. The globus major and minor are more affected than the body, and the former generally enlarges before the cord.

* Our author has seen a healthy boy, between twelve and thirteen years of age, affected, without obvious cause, with inflammation in the left testicle.

The latter becomes enlarged and tender, and, being compressed at the ring, gives rise to much uneasiness at that spot. The cremaster is in some cases affected with spasm. The scrotum is thickened and red, it pits upon pressure from effusion into its cellular texture, its veins are enlarged, and bleed freely when opened.

The symptoms take many days to arrive at their height, and still longer to disappear; evil consequences being frequently produced, which ever afterwards remain. If gonorrhœa be the cause, the urethral discharge usually stops or is considerably diminished, but generally returns as the inflammation subsides. During the violence of the complaint, the system often suffers severely from fever, and the blood, if drawn, is buffy.

Acute inflammation of the testis, when from sympathy with the urethra, rarely proceeds to suppuration; when from a blow or atmospheric changes, suppuration does occasionally occur, all the symptoms being aggravated and rigors superadded. The matter too is confined within the tunica albuginea, which does not readily give way; the pus is therefore long before it discharges itself after fluctuation is felt. The abscess generally breaks at several apertures, which form sinuses difficult to heal, and give issue to a seminal as well as a purulent discharge.

The diagnosis of this affection is seldom difficult, the only complaint with which it can be confounded is hernia. Still it is only congenital hernia that it can resemble, for, in common inguinal hernia the testicle can be readily distinguished. The history of congenital hernia, its long continuance, and its frequent descent from and return into the abdomen, contrasted with the gradual approach, excessive hardness, and severe pain in the loins, accompanying inflammation of the testicle, will serve as distinguishing signs. In a case of Mr. Samuel Cooper's in which the symptoms were so severe as to simulate strangulated hernia, the want of a particular protrusion at the ring, the absence of tension of the abdomen, the pain being confined to one side of the belly, and its not being increased on pressure, were the diagnostic marks. If a hernia had previously existed on the same side; if there be a swelling attended with exquisite pain, sickness, and vomiting, redness or a purple colour of the scrotum, constipation, and abdominal tenderness, then much caution is required both in treatment and diagnosis. It is best to give an aperient forthwith and a purgative injection to determine the question. The swelling will be harder than hernia, different in form, and more painful.

Hæmatocele may be mistaken for inflammation of the testis. The swelling rapidly following the blow, the ecchymosis usually present, and the comparatively little pain will distinguish it. We remember a case which appeared to be an urgent one of hernia. By the application of æther a small portion of gut was returned, but the bulk of the swelling still remained, and proved to consist of hernia humoralis and varicocele, a puzzling combination.

The causes of inflammation of the testicle come next under consideration. The most frequent is irritation in the canal of the urethra, and especially the membranous and prostatic portion, rarely the first six inches. In the early stages of gonorrhœa it is uncommon, most frequent when from ten days to three weeks have elapsed. The reason is, that when gonorrhœa has existed so long, the inflammation may extend from the first three inches of the urethra to the membranous portion. Thus in a person labouring under gonorrhœa, who was executed at the Old Bailey, the mucous membrane of the membranous portion was found inflamed, and even blood extravasated beneath it. Sir Astley, however, has seen cold water injected into the urethra induce a swelling of the testicle, shewing that irritation of the early part of the canal can do so. Our author believes that the hernia humoralis is brought on by extension of the inflammation along the vasa deferentia, and he thinks that both testicles not being affected is no objection to this position. He thinks that disease of one testicle diminishes the tendency to inflammation in the other, but this is perhaps problematical. It is not, as already explained, the most acute inflammation in the urethra, which acts upon the testis, but usually its declining stage, when the inflammation is rather extensive than violent, and of the erysipelatous character.

Astringent stimulating injections often produce inflammation of the testis, by lessening the urethral discharge. When injections are employed the patient should compress the urethra two inches from the orifice, a precaution which we find strangely neglected in practice. Bougies introduced into the membranous and prostatic portions of the urethra, often produce inflammation of the testis, and caustic bougies introduced so far have often the same effect. Instruments are often used unnecessarily, the obstructions resulting from temporary irritation ceasing under a merely soothing plan. A gentleman used a strong injection, which he thought produced a stricture; a bougie was passed, produced inflammation of the bladder, and was abandoned. In a year and a half afterwards he discharged a quantity of blood from the urethra, and two attacks of hernia humoralis succeeded. He has since been free from any obstruction. Any injury to the prostate has the same effect, as sometimes happens after the operation of lithotomy. A high legal character suffered much from this cause. The enlarged prostate of old age is occasionally attended with inflammation of the testis. Inflammation in the neck of the bladder produces the disease, as does a calculus in the bladder pressing on the orifice of the urethra, or one passing down the ureter, though the latter usually produces only spasm of the cremaster.

A blow on the testis is a frequent cause. If severe, vomiting instantly follows, and violent inflammation immediately succeeds. A person struck the testicle, next morning he could not make water, and when it came, by fomentations, it was tinged with blood. From that time the testis has been inflamed, and he has had a discharge of pus and blood at times from the urethra. A wound of the testis does not occasion the pain or inflammation which might be expected. Sir Astley has known a lancet and even a trocar thrust into it, a sickening pain followed, but the wound healed readily. In one case, however, violent inflammation and suppuration succeeded such a trocar-thrust.

An undescended testis is frequently exposed to injury and inflammation. Mr. Pott has detailed a good case of this kind, to which Sir Astley alludes. Wearing a truss on such a testicle, from mistaking it for hernia, or its being accompanied with hernia, produces great pain and inflammation. The surgeon must be careless in not observing the absence of the testis from the scrotum.

Vicissitudes of temperature affect the scrotum like other parts, and, by sympathy, the testes inflame. In a person who was in the retreat of the Duke of York's army in the Netherlands, the scrotum was frost-bitten and sloughed away. The sudden change of dress, from a warm to a very slight one, produces inflammation of the testicle. Sponging the parts with cold water, or going into the cold bath whilst the body is heated, are followed by the same effect. The excitement of the passions, with incapacity to indulge them at the moment, occasions great pain, and, from the distention of the tubes and unyielding nature of the tunica albuginea, is apt to give rise to inflammation.

The short section on the effects of inflammation of the testis cannot be much abridged; we therefore give it in Sir Astley's own words.

"An effusion of serum into the tunica vaginalis is a frequent effect of inflammation of the testicle; but this species of hydrocele usually becomes absorbed as the inflammatory attack subsides. The second effect which it produces, is adhesion and thickening of the tunica vaginalis, which is often mistaken for a disease of the testicle itself. This adhesion of the tunics is a very frequent consequence of inflammation of the testicle; and I have found, on examining testicles which felt harder than usual, that one surface of the tunica vaginalis was glued to the other, in some cases partially, and in others entirely, by which the mobility of the testicle in the scrotum had become diminished, and it less easily eluded pressure and external violence.

A swelling of the epididymis is a third effect of inflammation, which is sometimes placed at its lower, and sometimes at its upper part:—when at the lower, it is seated in the cellular tissue of the vas deferens, where it forms its first convolutions; but it often is not an effusion into the interior of the duct, which the patient will be gratified to learn, as his mind is rendered anxious about its influence on the function of the part.—Often these indurations are the effect of adhesion in the tunics only. When seated in the upper part of the epididymis, in the globus major, adhesive matter is effused into the cellular membrane, between the coni vasculosi, at their termination in the epididymis; and sometimes a sac, containing a mucilaginous fluid, is found at this part.

This portion of the epididymis is more frequently diseased than any other part of the testis or epididymis; but the result is less important here than in other parts, because some of the vasa efferentia and coni vasculosi still carry the semen from the testicle to the epididymis.

The coni vasculosi under this state of disease are thickened, hardened, and of a dark brown colour: in six testicles which I received at one time for dissection, of old persons, four of them were thus changed.

I have also a preparation in my possession, in which, after inflammation, a tumour, somewhat larger than a pea, was seated amidst the seminiferous tubes of the testicle, surrounded by an extremely vascular surface; and the testis was larger than natural.

In general, I observe that where there are marks of inflammation upon the tunics of the testis—such as, for example, adhesion—the substance of the gland itself is changed, the septa are much more apparent than natural, the seminiferous tubes appear to be less in number, are undoubtedly much reduced in their size, and many become cords instead of tubes." 23.

Wasting of the testicle, another effect of inflammation, requires consideration. It is more common at the age of puberty than any other time; it may follow a blow, occur spontaneously without obvious cause, or, though rarely, be the effect of gonorrhœa. The testicle inflames and swells; then, as the inflammation subsides, absorption commences, but does not stop till the whole of the glandular structure is absorbed, leaving the tunics adherent to each other and to the septa. The whole substance which remains is not larger than the end of the finger, and feels a firm and very solid body. In a wasted testis at St. Thomas's Hospital, quicksilver would only descend in the vas deferens half way between the ring and testis. A gentleman, aged 19, struck the testes on the pommel of a saddle. Acute inflammation in one testis followed, and when this subsided it wasted. The spermatic cord and the vast deferens were much diminished in size; a small portion of the epididymis could be felt; the testis was no larger than a pea, when swollen by moisture, and less sensitive than natural. The habit was scrofulous—the virile powers were not diminished. A gentleman, whose testis was wasted to a small hard body, felt constantly pain in the part, if unwell from a cold or other cause.

These facts prove conclusively that persons *should* be upon their guard in avoiding the causes, or, if excited, carefully attending to attacks of inflammation of the testicle. They will *not* be on their guard, for all that Sir Astley can say. The important subject of treatment now comes upon the tapis.

The first and most important measure is suspension of the scrotum by means of the bag-truss or handkerchief. If the former is used, the two anterior tapes should be carried to the loins, where they are to cross and be tied on the fore part of the abdomen; whilst the two posterior ones should be brought up to the fore part of the groin, upon each side, and be fastened to those which surround the abdomen. If the hinder tapes are carried between the thighs as they usually are, they are worse than useless. If a handkerchief is employed, a piece of tape should be added to the middle of its base and carried between the thighs to the back, where two of the angles of the handkerchief are to be tied, whilst the third angle is brought forwards and upwards before the scrotum. A lotion, composed of liq. plumb. acet. dil. ℥vij. sp. vin. ten. ℥j. is extremely useful. Vinegar or the liq. ammon. acet. expose the patient to observation by their scent. The muriate of ammonia, one drachm to a pint of water, is an excellent application, and has no smell. Active aperient medicine is required, and by these means the more acute inflammatory symptoms are removed. If these continue, leeches must be applied, or, if the patient object to the exposure they occasion, he may stand before the surgeon, who punctures three or four veins of the scrotum with a lancet, introduced transversely with respect to them. The bleeding will be free, especially if the parts be placed in warm water. As soon as three or four ounces of blood are drawn, he should recline upon a couch, when the bleeding almost immediately ceases. The recumbent posture must be insisted on for obvious and very sufficient reasons; the scrotum, however, being still supported. After the preceding means have been adopted, the relaxation and perspiration produced by fomentations and poultices are the best applications. The poultices should be thin, but their material is of little consequence. If the inflammation continue, local bleeding must again be had recourse to. Sir Astley has several times seen it absolutely necessary to bleed from the arm. Emetics in this stage of the disease are very useful, and nauseating doses of tartarized antimony are powerful adjuvants to treatment. In some irritable habits the continuance of depletion will prove injurious, and in such, when the pulse is jerking, calomel and Dover's powder often succeed when other means will fail. When suppuration has taken place, we must continue the fomentations and poultices, and leeches will lessen the extent of the suppuration. As soon as matter can be perceived it should be discharged by a lancet, as the tunica albuginea is long in ulcerating, and the secreting substance of the testis will be destroyed. Frequently the abscess is in the testis, often in the epididymis; more than once Sir Astley has seen it in the cord. The opening must be sufficiently free for the easy escape of the discharge.

The next object that engages the attention of the surgeon is the removal of the effects of inflammation of the testicle. The local applications for the enlargement and thickening left behind are poultices of vinegar and oatmeal, or the ammon. mur. lotion, with vinegar mixed with bread. The cerat. saponis, or linim. hydrargyri, or iodine ointment are also useful. The umbrella oil silk is an excellent application, promoting free secretion and unloading the vessels; the suspensory bandage may be formed of oil silk or lined with it. The emplast. ammoniac. c. hydrargyro is useful, the tincture of iodine may be applied, and the pyroligneous acid is a powerful irritating application.*

The best constitutional treatment is to give small doses of the oxymuriate, or pil. hyd. gr. iij. ant. tart. gr. ¼; or ext. col. c. gr. iij. with pulv. ipec. gr. ij. in a pill each night. If it nauseate, so much the better, as nausea promotes absorption. The liquor potassæ is a good medicine. Sir Astley has seen the enlargement of the epididymis yield to a three-months' course of the Plummer's pill and sarsaparilla. We may state, that the combination of the liquor potassæ and sarsaparilla is of service in strumous habits, and we have witnessed benefit from a solution of the hydriodate of potass, in conjunction with the steel wine. The tincture of iodine is useful, but it must be carefully watched, as it is apt to disorder the health. In obstinate cases, Sir Astley proposes the exhibition of the tincture of digitalis, but he believes that the promotion of nausea is the most powerful of all agents on the absorbent system. Sir Astley has not witnessed much advantage from electricity. The fact is, that these enlargements, especially of the epididymis, too often resist every medicine or application, and yield, at length, with the perfect and permanent re-establishment of health. Sometimes, indeed, they never yield at all.

Adhesions of the tunica vaginalis are not of sufficient consequence to justify active measures. If irritation or stricture of the urethra have been the cause of inflammation in the testis, bougies may be employed when the latter is removed, but not before. This concludes the chapter on hernia humoralis, a very important and useful one, because the disease is of frequent occurrence, generally tractable if treated well, followed by many bad effects if treated ill.

### Simple Chronic Disease of the Testicle.

Chronic inflammation of the testis is of very frequent occurrence, has of-

---

* A mixture of the unguentum potassæ hydriodatis and unguentum hydrargyri fortius, half an ounce of the former to an ounce and a half of the latter, is extremely useful. It usually produces some vesication.—Rev.

ten been confounded with malignant affections, and has repeatedly given occasion for an unnecessary castration. This is sufficient to impress on the minds of our readers the necessity of studying it with care. It begins in a hardness and swelling of the epididymis, at first unattended with pain, and discovered by accident. Gradually increasing, the testis becomes involved, but its separation from the epididymis may be distinctly traced. The testis usually retains its natural smoothness of surface, but is preternaturally rounded in form. Clear serum is, in many cases, effused into the tunica vaginalis; the part is indolent, free from pain, and handled with surprising roughness by the patient. The health appears to be unaffected, but in this, as in most chronic diseases, there is something out of order. Each epididymis and testis are often contemporaneously affected; hydrocele often exists on one side and not on the other, occasionally on both. One testis will cease to swell, and the other then become enlarged. The testicle and epididymis continue smooth under great increase, and the cord is not usually hardened, but its veins are a little swollen, and, consequently, it is somewhat increased in size. We have seen the cord enlarged and thickened in one or two cases. When the enlargement in the testis and epididymis is considerable, slight pain, and a sense of weight in the loins and thigh are complained of. Such is the history of the disease, very well detailed by Sir Astley Cooper. We have not found it follow this course invariably. We have seen much more pain complained of; the affection has not always begun so unequivocally in the epididymis; the distinction between this and the testis has not been so manifest at a more advanced stage; the surface of the latter has not been so uniformly smooth and regular, but more disposed to be knobbed and uneven. We should say that, in the majority of instances in which thickening and induration are left after acute inflammation, the epididymis is affected; but, in the slow chronic disease described by Sir Astley Cooper, we are disposed to doubt if this be so generally the case.

In the state above described by our able author the gland remains for months, receiving little other attention than support. From catarrh, a slight blow, or an excess, it becomes additionally swollen, attended with great pain in the part and loins, and swelling and redness of the scrotum. These symptoms are relieved by leeches and purging; but, in a few weeks, the return to exercise and the usual mode of living induces a relapse, requiring similar treatment. A repetition of these attacks so wearies the patient, that he wishes for castration.

At length suppuration is induced, and is indicated by great pain, redness of the scrotum, and obscure fluctuation. The abscess is felt at the extremity of the epididymis, or in the testis; if punctured, thick, ill-formed pus is discharged. If formed in the body of the testis, the tunica albuginea retards greatly its progress to the skin. One or more sinuses are produced, giving issue to seminal discharge, which retards their healing, or prevents it. During the progress of the suppurative inflammation a hydrocele is formed, and the fluid is generally coloured by the red particles of the blood.

A prominent object of attention is the granular swelling of the testis, which arises from the cavity of the abscess, whether of the epididymis or testis. Exuberant granulations spring up, and as they grow, being compressed by the albuginea, they protrude through the ulcerated opening of the tunic and form a granulated swelling, often seen on the scrotum.

It resembles in principle the fungus cerebri. It has been mistaken for cancer or fungus, but it may be cured by local applications, and is in no respect malignant.

Sometimes this disease will really require removal, from the continued irritation and discharge which it produces, or from its great inconvenience. When removed and dissected in the adhesive stage, the appearance of the testicle and epididymis is changed into a general yellowish white appearance, possessing considerable solidity. It was this, we believe, that has induced Mr. Brodie to name the affection, the yellow tubercular disease of the testicle. If a section is made of the testis, thrown into water, and agitated, a whitish yellow fluid proceeds from the seminiferous tubes, which appear emptied and are much dilated. But still the same bulk remains, owing to the cellular membrane being loaded with yellow fibrine. The rete is filled with the same secretion as the tubuli, the epididymis is similarly diseased, and sometimes the vesiculæ and vasa deferentia are distended with a similar morbid secretion. But the effusion, whether in one or the other, admits of being absorbed by proper treatment, and appears to leave the testicle capable of performing its functions. Sometimes we meet with an abscess or abscesses in the testicle and epididymis, connected with the adhesive or fibrous effusion; and this is combined with more or less of ulceration, destroying a part of the testicle, and rendering complete recovery of its functions impossible. Several abscesses are sometimes found in the same testicle. In the third place, we find sinuses externally leading to these cavities. Still secreting semen, the cavities and their outlets are prevented from closing, till the secreting surface be healed or destroyed. In the fourth place, when the granular swelling is produced, the granulations are found to spring from the seminiferous structure, and project through the ulcerated opening of the testicle or epididymis, most frequently of the former. These points being ascertained, much light is thrown upon the treatment to be adopted by the surgeon.

With respect to the causes of the disease, Sir Astley believes that there is a constitutional proneness to this complaint. It occurs in the scrofulous, the debauched, after long mercurial courses, and in the debilitated habits in which we find chronic carbuncle. Frequent exposure to wet, cold, or fatigue, and excessive sensuality, are predisposing causes. The most frequent occasional cause is urethral irritation or organic alteration, and many of the causes of acute inflammation of the testis are, in different cases, the precursors of this affection.

We arrive at the treatment. The disease is not malignant, the lumbar or inguinal glands never being seriously affected. The great efforts of the surgeon should be directed to the adhesive or early stage, when fibrous matter only is effused, for we have seen that it is removable, and the organ capable of recovering its functions. The patient should steadily observe the perfectly recumbent posture for a month, for it will not be enough to sit upon a sofa with the legs supported. He is to take calom. gr. iij. opii, gr. j. night and morning, till the gums are affected, which they should continue to be for a month at least. Every fourth morning he should take a black draught, with liq. ant. tart. ℳ. xx. The local treatment should consist of leeches to the scrotum twice in the week, fomentations thrice daily, and a lotion of liq. ammon. acet. ℥v. sp. vini, ℥j. or equal parts of camphor mixture and water. By this plan, perseveringly followed for a month or six weeks, the disease rarely fails to yield prior to the suppurative stage. When the disease is sympathetic with the urethra, it will be right to use bougies, if the stricture be considerable, before the patient quits the recumbent posture. If the urethra be merely irritable and the stricture slight, it will be better not to use bougies, as the constitutional treatment for the testis often relieves the urethra, without any risk of aggravating the local disease. We think Sir Astley has omitted one remedy, mercurial inunction on the scrotum. It often aids the operation of the calomel and opium constitutionally, and appears to possess a local influence also. In bad habits the sarsaparilla and oxymuriate are preferable to the calomel and opium. The blue pill is likewise more adapted to some cases, but the principle is the same in all.

Sir Astley adds three cases illustrative of the foregoing positions;—we shall briefly notice two. A British officer of rank was attacked with inflammation of the testis in the Peninsula. He suffered much from fatigue, the disease was obstinate and thought to be malignant, and the testis was removed. Some time afterwards the other testis became similarly affected, and a consultation of Mr. Rose, Sir E. Home, and Sir Astley was called. They agreed to the plan already detailed, and in a few weeks the officer perfectly recovered. It is fair to suppose that the other testis might likewise have been saved.

If once suppuration, no matter how little, has occurred, the symptoms may appear to yield, but they return so soon as the patient rises and gives up mercury. Thus, a cavalry-surgeon had chronic inflammation of the testicle, which had often been relieved by treatment, yet returned when he resumed his duties. Tired out, he requested Sir Astley to remove the part, which he did. In the centre of the testis was a chronic abscess.

In the treatment of the granulations it is an object to reduce their exuberance, and bring them level with the skin. Pressure by dry lint and adhesive plaster bound round the scrotum effect this purpose. If this does not succeed, powdered sulphate of copper daily sprinkled on the surface may, or powdered alum, or the nitrate of silver. We have seen the powdered nitrico-oxyd of mercury very serviceable. Sir Astley has known arsenic thus applied destroy life, and the same placed in solution on a fungous disease of the eye, produced fatal inflammation of the stomach. Sir Astley has several times excised the granulations with success. He makes an elliptical incision around the projecting granulations, and then carries the knife under the whole, and close to the tunica albuginea, excising the fungus, and leaving the epididymis and testicle uninjured. The edges of the skin are to be brought together, and made to unite if possible. Union may be aided by pressure with adhesive plaster, and approximation of the integuments over the opening in the tunica albuginea. The operation must of course be preceded by proper constitutional treatment.

Our author has adopted another proceeding on more than one occasion, with success. He passed two ligatures through the edges of the skin at the circumference of the swelling, and carried them through its base. He then cut off the granulations even with the scrotum, and brought the edges of the skin over the new surface. If, however, the swelling be very large and the testicle much reduced, it is better to remove it at once. For the troublesome sinuses following abscesses in the testicle or epididymis, calomel and opium, with the recumbent position, are the best constitutional remedies, injections of the sulphate of copper or the oxymuriate of mercury, the best local ones. In an obstinate case, in which the abscess began in the globus minor of the epididymis, a deep incision was successfully made to divide the vas deferens there, prevent the continued secretion of semen from the orifice, and lead to the closure of the vessel. In one case the seton was employed. There had been abscesses and were sinuses in the epididymis of both sides: a gentleman passed a seton through each opening, and they both healed. In coition he has the sensation of emission, without it actually occurring. The testes are nearly of natural size, there is a hard swelling in the globus minor of each epididymis. When the operation of removing the testis is performed, the patient is free from future danger, with the exception of the vesiculæ seminales being diseased. This is rare, and, by improvement of the constitution, may be dispersed.

### THE IRRITABLE TESTIS.

This is a most distressing disease, and like other affections of the nature of tic douloureux, it is extremely difficult to cure. The patient has an unnatural sensibility in a part of the testicle or epididymis, which is excessively tender to the touch, and painful upon exercise.

"Its sensibility becomes occasionally so much increased, that the slightest touch produces exquisite suffering: the pain is felt in the back and groin. The motion of the testis, and the slight pressure it receives from the clothes in walking, produce so great a degree of pain as almost to forbid exercise; and the patient is obliged to seek relief, by continually reposing upon a sofa, or by remaining in bed. The testicle is but little swollen; it is not equally tender in every part, but there is a point in which the morbid sensibility particularly resides. The epididymis and spermatic cord also suffer from similar sensibility, and if the part be not supported, the pain is scarcely tolerable; and when the patient is in the recumbent posture, he is obliged to place himself on the opposite side to the disease, or he does not rest. He has pain in the groin and thigh upon the same side, and the testicle appears fuller and more loaded on that side than the other. Motion, in most cases, produces not only pain at the time, but much increased inconvenience for some hours after; the pressure of the hand in examining it occasions great uneasiness, and leaves the testis additionally sensitive. The stomach is rendered extremely irritable, even to the degree of occasioning vomiting." 50.

It may continue for weeks, months, even years; and if at any time a temporary relief is experienced, a slight indulgence or return to exercise renews the patient's sufferings. Sometimes the misery and inconvenience are so great, that the patient insists on the removal of the part, which Sir Astley has performed in three instances. To supply a more graphic description of the malady, our author inserts some letters from patients who were tormented with it. We shall give a quotation from one, which appears to be written by a medical man.

"'Probably this disease is seated in the nerve of the spermatic cord, and perhaps also in the plexus which surround the arteries; in which case you know the operation could not be of much service. To this conclusion I have the more decidedly come, from the circumstance that the pain is of a numb pricking kind, answering to that proceeding from a compressed or irritated nerve, and that it is uniformly increased by whatever disturbs the position of the testis, or presses upon the ring, or the course of the cord. I can bear the erect position for a short time, that is, for a few minutes, without my suffering being much increased, provided the testis is properly adjusted, and there is no pressure or irritation applied to the iliac or pubal regions of the right side. When I lie on the left, the pain is of the dragging kind, as if extending from the region of the cæcum coli towards the part where the injury on the cord was inflicted, as it crosses the pubis; whereas, when on the right side, it is more sharp, and feels as if some part which is tender and sore, were pressed upon by those in its neighbourhood. I feel most ease, therefore, when I lie on my back, the testis being well adjusted, and pressure of the bandage removed completely from the side. There is considerable fulness or thickening on the side of the pubis in the site of the injury, which is always increased, and extends higher up in the direction of the cord, when the pain has been greater for a little time; but the fulness and tension in the right iliac region, upon the whole, has been less for two or three months.

After medicine, which has by its action produced two or three stools, I have uniformly suffered more pain for a day; and the passage of the flatus through the cæcum is attended by a somewhat similar effect, though of much shorter duration. On examining the cord as accurately as the tender state of the part will allow, it appears to be free from disease; and the testis, which, however, hangs lower than the other, excepting its greater delicacy on being handled, seems to remain unchanged in size and structure.

My general health continues good, and every function natural.

My urinary organs are perfectly healthy; and I may remark that when the penis is erected, I am much freer from pain, probably in consequence of the contracted state of the scrotum, that takes place at that time, supporting the testes. This is a crude and hasty sketch; but from it you will be enabled to collect the particulars for your own arrangements.

The distance of time between the accident and my beginning to suffer much, with the exception of an occasional stounding once or twice in a month, was about eighteen months; and the length of time I have now been confined, always entirely to the horizontal position, is more than a year.'" 55.

In another case the individual thought he could trace back the origin of his disease to about eight or nine months before his marriage. During that time he lived too well, grew corpulent and bloated, took little exercise, and did not cohabit with women as he had previously done. Before the complaint began he had violent erections during his hours of rest, when the testicle and vessels felt ready to burst, and obliged him to get up and walk about for some time. Soon after his marriage the symptoms commenced, and in a few months he experienced some pain in discharging the testicle, which at last arrived at an alarming height.

We pass to the cause of the disease, a subject involved in some obscurity. It is clearly not inflammatory, for of inflammation there is no symptom. Sir Astley believes that the disease is seated in the nerve, and that it is of the nature of tic douloureux, in which the action is allowed to be generally below rather than above par. Mr. Thomas, the President of the College of Surgeons, dissected a suborbitary nerve affected with tic douloureux, but found nothing unusual in its structure. This nervous affection is sometimes local, as in an amputated limb; sometimes constitutional: sometimes sympathetic with disease of the brain and its membranes, as in the well-known case of Dr. Pemberton. In the irritable testis dissection teaches nothing, except that it is not inflammatory, for in three testicles examined by Sir Astley, he found no change of structure. The digestive functions are often impaired, but our able author looks on this as the consequence, not the cause, of the derangement of the nervous system; it is not permanently relieved by diet.

*Treatment of the Irritable Testis.* The surgeon's objects are to increase the tone of the nervous system, and to allay the constitutional and local irritability. Various medicines have been recommended and are employed with the first view:—quinine in large doses—bark—the carbonate of iron—the liquor arsenicalis—ammonia in large doses, combined with camphor. Wine, brandy, and other spirituous drinks relieve the severity of an attack, but tend to its renewal and increase. The second object is to allay the irritability of the nervous system. For this we have the various narcotics, of which a good form is, conii, gr. iij. opii, gr. j, extr. stramonii è seminibus gr. ss. bis terve die. Belladonna, from gr. ss. to gr. iij—hyosciamus—the black drop, and other preparations of opium—and calomel, opium, and antimony in combination, if the secretions of the liver and skin be defective. The local application of belladonna* in the shape of a plaster has been occasionally useful; so have opium and camphor, rubbed upon the part; ice has sometimes produced a cure. Applying a blister to the groin and thigh in the neighbourhood, and maintaining a discharge by the ceratum sabinæ cum opio is of service—irritation of the skin by the tincture of iodine has been tried with good effect—and the pyroligneous acid on the scrotum may be used, but it requires care.

When the complaint arises from organic disease of the brain, mitigation of symptoms is all that can be expected, but altered action in a nerve or in the nervous system generally admits of cure. A sea voyage to a warm climate has been known to give great relief.

It is generally Sir Astley's plan in this disease to begin by giving calomel and opium, so as slightly to affect the salivary glands and excite all the secretions; to these the sarsaparilla may be added, as it lessens irritability. He applies a blister to the groin, and keeps up the discharge by the ung. hyd. and cerat. sabinæ in equal quantities; and to the testis itself an evaporating lotion of diluted spirits of wine and æther, or of the nitrate of potass with muriate of ammonia. A slight discharge from the commencement of the urethra, produced by the unguentum lyttæ is at times of use. But in spite of all that our art can do there are cases which will not yield, and in which the patient insists on the extirpation of the part. Of these Sir Astley details three. In all the relief was complete and permanent, but in the first the wound healed slowly, and one or two small abscesses formed in the scrotum.

### INFLAMMATION OF THE TESTIS FROM CYNANCHE PAROTIDEA, OR MUMPS.

The cynanche parotidea is produced and accompanied by a species of

* One part of the extract to about five of ceratum saponis, spread on leather. If stronger it is apt to produce poisonous effects on the system.—REV.

fever the effect of which is a swelling of the parotid, and sometimes of the submaxillary and sublingual glands, occasionally accompanied with an enlargement of the mamma in one sex, and testis in the other. The disease principally affects the young, and the glandular enlargements are sometimes seated on one side, now and then on both. The disease reaches its height in a few days, then begins to decline, and in a few days more it usually disappears. Suppuration is rare either in the parotid, the mamma, or the testis. In the first case seen by Sir Astley, both the testis and epididymis were enlarged and tender, but the patient soon recovered. In another case each epididymis and testicle were enlarged, and of pyramidal form, owing to the epididymis being principally implicated; the scrotum was red, and the enlargement of the left side exceeded that of the right. This gentleman had formerly suffered from hernia humoralis, and the testicle continues swollen, although the fever which preceded and produced it occurred a month ago. The mumps in children are most infectious. In a school at Hackney, consisting of from thirty to forty, rather more than thirty were affected with the disease within the space of three weeks, but in no instance was there a metastasis to the testis.

When the testicle inflames in cynanche parotidea, it generally happens about the age of puberty, seldom before that, occasionally so late as between forty and fifty. The swelling of the salivary glands and the enlargement of the testes are generally proportioned to the severity of the cynanche. The affection of the testes is more frequent than that of the mammæ. Why the testis should be affected we do not know, as its structure differs entirely from that of the parotid. It has been said to waste after this complaint, but our author has never known it do so, though inflammation from any cause, at the age of puberty, will produce that effect.

The treatment is very simple; the liq. ammon. acet. with the sulphate of magnesia, salines with antimony, or calomel pill and antimonial powder. Leeches are proper, and a simple poultice should be applied to the neck; the best lotion to the testis is the liq. ammon. acet. and sp. vini. Sir Astley dreads the use of evaporating lotions on the neck, as he once saw them produce fatal symptoms of pressure on the brain, in a boy about eleven years of age.

### VI. Hydatid or Encysted Disease of the Testicle.

This is a comparatively rare disease, of a specific kind, and, as it appears to Sir Astley, entirely local, as he has seen it in persons enjoying excellent health, who have retained that health after removal of the testis, and in whom the disease has never shown itself at any future period. It is chiefly seen at about adult age, or between eighteen and thirty-five, although he has known it occur at forty-nine. It has been said to begin in an enlargement of the epididymis, but it is usually discovered by accident when it has attained considerable bulk. Sir A. has certainly several times seen the end of the epididymis containing cysts filled with fluid. It is unattended with pain, until it has become so large, that the unyielding albuginea exerts great pressure on the contained gland. There is little tenderness on pressure, unless it be considerable—the natural form of the testis is preserved, being rounded before, flattened on the sides, and not so pyriform as an hydrocele

—the epididymis is usually distinct but not always so—the veins of the cord are enlarged, and so are those of the scrotum. The swelling gives, on handling, the sensation of fluctuation, but this is deceptive, as the part yields only at the spot which is compressed. If the tumour be strongly pressed, the patient feels as if the testicle were squeezed. The weight of the organ is obviously augmented, and when its size is much increased, there is pain in the loins, and inconvenience from the bulk. The appearance of the individual often indicates even robust health, and therefore the first impression on the surgeon's mind is, that this disease must be hydrocele.

The complaint is so local, that, but for the irksome weight and the indecent and inconvenient size, it would scarcely require removal. Sir Astley declares that the cord does not become affected, nor are the absorbent glands of the groin or loins irritated by it; in short it is a disease of the testis and epididymis only. The cysts connected with fungoid disease, differ entirely from this hydatid complaint, for they contain a fungous growth within them. We are afraid that Sir Astley has overrated the mildness and local character of this affection. In a case at St. George's Hospital, in which castration was performed by Mr. Brodie on a testicle exhibiting the hydatid disease, death from peritonitis ensued, and an opportunity was afforded of examining other parts. The lumbar glands *were* affected in a similar manner with the extirpated testis. Again, it is by no means uncommon to find cysts, containing no fungus whatever, in testes and breasts contaminated by fungoid disease.

*On dissection of the hydatid testis*, the tunica vaginalis is thickened and partially adherent; the albuginea universally denser than natural. The testis is composed in part of a solid structure and in part of cysts, varying in size from the head of a large pin to the magnitude of a small marble. The small contain a serous fluid, transparent and yellow; the larger have undergone a change from inflammation, their coats being thickened and their contents mucous. The cysts containing serum are highly vascular, but those which have been inflamed cease to be transparent.

"The appearance of the testicle would indicate that the cysts are enlargements and obstructions of the seminiferous tubes, thus increased by the accumulation of fluid within them, and connected with each other at their different convolutions; but their origin must be in a great degree conjectural, whether they be produced in the cellular tissue, by effusion into its cells, or in the seminiferous tubes.* They certainly are not of the nature of animal hydatid; but I am inclined to the opinion, that they are formed of enlarged and obstructed seminiferous tubes; for when I minutely dissect them, although at first sight they appear to be cysts, yet when traced, they are not distinct bags, but send out solid processes, by which they are connected with other bags, as the plate will shew. It ought, therefore, to be called the *Tubular* Disease of the Testis." 83.

The appearances of the epididymis are similar to those of the testis, but the cysts are always smaller.

*Diagnosis of the Hydatid Disease.* It is often mistaken for hydrocele,

* "I have seen absorbent glands undergo the same changes from enlargement, obstruction of their cells and vessels, and from a fluid being secreted into them."

even by the surgeons of large metropolitan hospitals. Sir Astley, with that manly candour, which even the foul attacks of the slanderous publication that pollutes our profession have not yet been able to destroy, acknowledges that he has been on two or three occasions himself mistaken. He recommends that in all doubtful cases, a small incision be made with a lancet into the tunica vaginalis, in order to ascertain if it does or does not contain fluid. A better instrument than a lancet, is a fine grooved needle, about the size of a couching needle, but larger at the base. This makes a minute puncture, and fluid, if it exists, escapes along the groove. The general marks of distinction between the two diseases are, first—a yielding rather than a fluctuation;—secondly, a heavier tumour;—thirdly, the general form of the testis being preserved, although it is somewhat more pyriform than natural; —fourthly, the entire absence of transparency;—fifthly, the sensation of the testis being squeezed, if the compression be considerable;—sixthly, the dilated state of the vessels of the cord and scrotum;—seventhly, the testis in hydrocele can be felt at the lower and back part of the swelling, although obscurely.

*Causes of the Disease.* These are entirely unknown, although it is usually imputed by the parties to a blow or a cold. It appears to be in its nature an obstruction of, and altered secretion into the seminiferous tubes.

*Treatment.* Our author has never seen any medical or local treatment of the slightest service. The removal of the part is the only measure that can give relief, and a system of depletion and abstinence for a week previous to its performance, will enable the patient to bear the operation well. Sir Astley observes that the strongest assurances may be given to the patient, that it will never produce any influence upon the surrounding or adjacent parts, and that the operation will be completely successful in eradicating it. He has never known it unsuccessful. We refer again to the case to which we have already alluded, to shew that the circumstances are not always thus favourable. Four cases are detailed by Sir Astley. The following affords the best sample of the progress of the complaint.

*Case.* Charles Denby, aged 49, was admitted into Guy's Hospital on the 23d May, 1804, with enlargement of the testis, which had begun two years previously in a diminution of the part, attended with a sense of weakness on the same side. It afterwards gradually enlarged, and in nine months from its first increase he applied to a very respectable surgeon who introduced a trocar into the testicle. A very small quantity of water was discharged, and the case was pronounced to be hydatid testis. The disease increasing, the patient was admitted into the hospital, and on the 29th of May, Sir Astley removed it by the knife. On cutting into the testis there were cysts of various sizes, some transparent, others opaque: some filled with serum, others with mucus, and others again with a clear water, containing little animal matter. The wound quickly healed, and the patient was discharged cured on the 16th of June.

### VII. Animal Hydatid in the Testis.

Sir Astley has never seen this disease in the living subject, and therefore of the symptoms he knows nothing. Mr. Davie, formerly a dissector for St. Thomas's, brought our author a testis, the epididymis of which contained a cyst, formed by adhesion. Within the cyst was an hydatid, perfectly detached from the bag in which it was contained. The testis was larger than usual.

### VIII. Scrofulous Inflammation of the Testis.

Chronic diseases are founded either on an originally delicate and weakly formation, which we designate scrofulous; or on changes produced by anxiety, intemperance, &c. in an originally healthy frame: in fact, on debility, not congenital but acquired. The shortest idea which our author can give of scrofula is congenital or original debility, marked by peculiar and generally well-known signs. The skin is extremely thin and delicate, sometimes light and sometimes dark, but thin always. The blood that mantles on the cheek of the fair girl is considered by the looker-on as beauty; the physician sees in it the token of weakness, too often the herald of decay. This flimsy delicacy of integument also gives rise to the darkness beneath the eye, produced in such habits by slight indisposition, and dependent on remora in the veins. The thick upper lip depends on retention of blood in this vascular tissue; the thin skin shews the same vascularity in the tarsal glands. The hair is flaxen, or delicately silken, and red-haired persons are strongly predisposed to scrofula. But black hair and a dark skin, if the latter be thin, are evidences of the diathesis; indeed we have often been struck by the black fine hair, large black pensive eye, and delicately sallow cheek of strumous children. Bad air and bad food may give rise to serious chronic diseases in healthily-formed children, but then the complaints resemble more the chronic affections of adults.

By reason of the thinness of the skin, the cuticle chaps and desquamates from a blast of cold air, parches and cracks with the Summer's sun. Vicissitude of cold and heat excites inflammation of the cutis, the cutaneous absorbent vessels become irritated and the absorbent glands inflame. As the face and ears are most exposed, so the glands connected with their absorbents suffer readily; hence the frequent glandular enlargements in the neck.

When we look to the interior, we still find the same delicacy of structure and flimsiness of fabric pervading its component parts. The stomach and intestines are pellucid—digestion is imperfect. The parietes of the heart are less muscular than usual—the circulation is feeble. The coats of the arteries are so thin that the blood is clearly seen through them, in the last acts of life they do not empty themselves as under common circumstances, and their deficient tone must additionally weaken the circulation. The veins and absorbents probably partake of the same feebleness, at least the latter, and their glands are peculiarly prone to morbid action. The secretory glands, with the exception of the mucous glandules of the intestines, are little prone to scrofulous maladies. From the irritation and inflammation of the latter, the mesenteric disease is produced.

The nervous system differs greatly in different persons. In some, there is so remarkable an indolence and apathy, that a joint will enlarge without pain, and suppurate with scarcely any disturbance of the system. The mind, like the body, suffers no alarm. But in others there is a remarkable irritability. From the very dawn of the complaint there is severe pain, and the slightest bodily excitement produces extreme constitutional irritation. Metastasis occurs from joint to joint, the temper is fretful and anxious to a degree, the least irritation is felt as through a magnifying medium, and there is often a surprising precocity of talent. The fond parent looks with admiration on her offspring, and crowds study on study, accomplishment on accomplishment, till consumption, or some other form of the dire malady, snatches away the object of her pride and pernicious cares. The intellect is feverish, rather than healthy and powerful; its efforts should be moderated and repressed, rather than forced and encouraged.

The testis is one of the exceptions to the non-liability of secretory glands to scrofula. Even in very young children it sometimes becomes enlarged and very hard, without pain or inconvenience, the swelling being accidentally discovered, and remaining indolent for months or years, till it subsides with the improvement of the general health. More frequently it enlarges at the age of puberty, or from that to twenty years; often it appears in both testes without pain or tenderness, without discolouration of the scrotum or enlargement of its veins. But for its bulk the patient suffers no inconvenience. In children, and more frequently at the age of puberty, the inflammation proceeds to suppuration, generally in the globus major of the epididymis, sometimes in the globus minor. The body of the testis rarely suppurates, but, after ulceration of the epididymis, the testis becomes affected and the scrotum livid; ulceration ensues, and an abscess forms, which discharges ill-formed pus and some semen, at least after the age of puberty; and the opening continues unhealed for months and for years. In some persons, one abscess forms and discharges after another, and when one testis has suppurated, the other, if it has been hard, does the same, obstinately resisting all means of treatment for a great length of time.

At length the testes diminish till but little remains, and the seminal secretion ceases almost entirely. In a gentleman in whom both testes wasted, only two or three drops of thin fluid issued on emission, but he had erections and occasionally sexual desire. He had the disease for four years, and a sinus from each epididymis still discharges a quantity of fluid, which stiffens the linen. After venereal excitement, the sinuses give issue to an increased quantity of fluid, and in one patient it became of a brownish colour, as if slightly tinged with blood.

*On dissection* of the epididymis and testis, Sir Astley has found a yellow spot in it, surrounded by a zone of inflammation. When the spot ulcerates in its centre, the matter which it contains is not pure pus, but is composed of fibrine and serum, with a slight yellow tinge. Sir Astley has seen it in the globus minor of the epididymis, more often in the globus major. In the testis there are several such yellow spots, with surrounding inflammatory zone, and several yellow streaks are found amidst the tubuli. Scrofulous abscesses in the testes are sometimes accompanied by a granular swelling, like that which exists in the simple chronic disease.

*Diagnosis.* This disease is known by the period of life at which it occurs, by the patient's habit of body, and by the presence of scrofulous disease in other parts.

*Treatment.* We must invigorate the constitution by good air, especially that of the coast; exercise, avoiding riding on horseback; nutritious, unirritating diet; ale or porter, or wine and water at dinner, unless flushing and heat be produced by them. Tepid sea-bathing should also be used. Hyd. c. cret. with rhubarb on alternate nights, or pulv. calumbæ, rhubarb, and soda twice per diem; the vin. ferr. the tinct. ferr. mur. tinct ferr. ammon. or ferr. carbon. with pulv. rhei in pills, are very useful. The quinine, with infus. ros. and dilute sulphuric acid should be given, and the liquor potassæ may be tried, but its long continuance is apt to weaken the stomach. The oxymurias hydrargyri in small doses with sarsaparilla, or with tincture of bark and rhubarb, is an admirable medicine. Concentrated compound decoction of sarsaparilla is often advantageous; our author has seen such serious effects from the tincture of iodine, that he fears advising it. Thus many different medicines are applicable to different cases, but the principle in all is to restore the secretions and give tone to the system.

In the early stage of the disease the ointment of iodine may be rubbed upon the part, or the liniment. hyd. or the emp. hydrargyri, the object being to stimulate the absorbents. As lotions, the liq. ammon. acet. with sp. vini, or the liq. plumb. acet. with sp. vini if there be pain. A dilute sulphate of copper lotion, or the black-wash may be injected into the sinuses, if they form. Port wine may be used in the same manner. A solution of the oxymuriate has been useful, and our author has found the tinctura lyttæ or a solution of the nitrate of silver beneficial.

### IX. Venereal Inflammation of the Testis.

The venereal poison, when absorbed into the system, affects more particularly, and generally in order, the throat, the skin, and the periosteum, with the bone beneath it. The eye is more rarely affected, the testicle is the same, but the abdominal and thoracic viscera and the brain appear to be exempt from the influence of the poison. Some persons doubt the susceptibility of the testis to the venereal influence; but our able author has seen it so frequently enlarged during secondary symptoms, more especially in combination with cutaneous and periosteal affection—has seen it display the periodical evening exacerbations, although the recumbent posture was adopted at the time—and has known it yield so readily to mercury, just in proportion to the disappearance of the venereal symptoms, that he entertains no doubts whatever on the subject. The gonorrhœal affection of the testicle requires not the exhibition of mercury, it is sympathetic only.

Sir Astley believes that the venereal inflammation of the testis attacks the tendinous structure, the albuginea, and thence extends into its interior fibrous, not tubular part. He reasons thus, however, from analogy, for he has had no opportunity of dissecting this disease. The testis and epididymis enlarge to four or five times their natural size, and when one is attacked the other is apt to become implicated; indeed, in most instances, both are affected. The pain is not severe, but it is increased towards evening; the complaint very rarely proceeds to suppuration, but when it does, it produces a granular swelling, like the chronic abscess. The enlargement of the testis is rarely a concomitant of the syphilitic sore throat only; it frequently accompanies the eruption and periosteal inflammation. Its succeeding syphilitic symptoms, and being combined with those alluded to, as also its evening exacerbations, are the diagnostic marks of this disease. Eight cases are related; we can find room for one or two.

*Case.* A gentleman had a hydrocele with enlarged testis, and a surgeon, in attempting to tap the former, wounded the latter; he concluded there was a solid enlargement of the testis, and proposed extirpation. Sir Astley being called in found enlargement of the tibia with nocturnal pains, and a venereal eruption on the fore-part of the chest and abdomen. He ordered a course of mercury, when the syphilitic symptoms vanished, and the enlargement of the testis disappeared. The hydrocele was injected, the patient got well, has since been married, and had children.

*Case.* A gentleman, aged 32, had four years ago a chancre, for which he took mercury until it was healed. A few months afterwards he had pains in his limbs and his head, succeeded by enlargement of the tibia. He used mercury at various times, in sufficient quantity to subdue the symptoms, but not to cure the disease. Fourteen months ago a swelling began in the right testicle and gradually increased; then he suffered from pain in the left, in which a hardness remained. Sir Astley affected his mouth with calomel and opium, and continued it for six weeks. He made him observe the recumbent posture, apply leeches and a lotion of the liq. ammon. acet. and sp. vini. The swelling testis was entirely reduced, but when he left town the pain in his leg was not completely subdued.

Sir Astley is not inclined to dispute with those who doubt the syphilitic nature of such cases, but he is sure that he has seen enlargements of the testis combined with syphilis, and that the best mode of treating them consists in putting the system fairly under the influence of mercury, exhibiting afterwards a lengthened course of the compound decoction of sarsaparilla. He feels assured, in fact, that the testicle becomes affected during the progress and influence of the syphilitic poison upon the body in some persons, and that mercury is the only cure. At the same time we must employ the recumbent posture, local depletion, and evaporating lotions, although without mercury they will inevitably fail.

### X. OSSIFIC INFLAMMATION OF THE TESTICLE.

The deposit of earthy matter not unfrequently takes place in structures not originally containing it. It is most commonly seen in the cartilages of the larynx, trachea, and ribs. It is found in ligamentous structures, as the symphysis pubis, sacro-iliac synchondrosis, and ligaments of the spine; in serous membranes, as the arteries, the pleura, pericardium, and peritoneum; and in most instances it is the concomitant of old age.

In dissecting enlarged and much hardened testicles, our author has sometimes met with deposits of earth, variously situated. The tunica vaginalis occasionally undergoes this change, a beautiful specimen of which may be seen in the museum at Guy's Hospital. The tunica albuginea is still more frequently affected, little patches of earth being often seen between it and the tunica vaginalis testis; the albuginea is also sometimes entirely covered, as well as interstitially loaded with earthy matter. When a hardness is left by chronic inflammation at each extremity of the epididymis, earthy matter is sometimes found in the globus major or minor. The testis is less frequently affected, but when very enlarged, portions of cartilage containing earth are found amidst the recently effused solid matter. A simple chronic disease will occasionally, under a length of time and changes of the constitution, undergo such alterations that various appearances will be found in it—pulpy substance, cysts, cartilaginous and ossific matter.

*Case.* James Verroil, aged 26, a musician at one of the theatres, was admitted into St. Thomas's Hospital, on the 8th April, 1824, under the care of Mr. Tyrrell. His aspect was sallow, his secretions irregular, and there was much constitutional derangement. One testicle was about the size of a large orange, rather uneven on its surface, hard in some parts, soft and fluctuating in others. There was at times severe pain in the affected part, extending to the loins. In the Spring of 1823, he had contracted a gonorrhœa for the fourth time, which in three or four weeks gave rise to inflammation and enlargement of the testis. By rest and evaporating lotions the inflammatory symptoms were reduced, but the testicle still remained hard and much larger than natural. He returned to his former irregular mode of living, and in the following October, the testis enlarged still more, particularly at the posterior part. From that time till his admission the enlargement had continued progressively increasing.

The remedies for chronic inflammation being tried without success, Mr. Tyrrell removed the disease. On examination of the testis, its substance was converted into a soft pulpy or medullary matter, in the centre of which was a small abscess. The epididymis presented a hard mass like scirrhus, had numerous portions of cartilage deposited in it, and at its upper part was a bunch of hydatids. An attack of peritonitis followed the operation, but the patient recovered, and left the hospital cured.

These cartilaginous and ossific deposits, whether in the membranes or in the substance of the testis, admit of no relief from medical or surgical treatment. The operation of castration is not generally required, as they remain for many years in an indolent state, and urgent measures are unnecessary, unless the complaint assume a malignant disposition, or be very inconvenient from its bulk. Sir Astley believes that such deposits are more frequently the effect of long-continued simple chronic inflammation, and of change of structure from age, than the consequence of malignant action in the part.

This concludes the portion of Sir Astley Cooper's magnificent work, which treats of the simple diseases of the testicle, a portion comprising 115 pages. We have placed within the compass of a little more than twenty, almost every individual observation and precept of the observant, experienced, and able Baronet. It has required no talent to do this, it merely needed dense and unwearied analysis. We have added few remarks of our own, for although the cases we have witnessed might justly enable us to say more, we thought that the most useful boon to our readers, would be placing before them the sentiments of Sir Astley Cooper, without the alloy of trite observations or specious criticism. Little summing up is required, for, in the present case, any censure would be absurd, and praise superfluous. We are not presented with flimsy theories, which would probably be upset by the visionary of to-morrow, but with solid precepts deduced from long and careful observations of the book of Nature. The malignant diseases of the testicle, and the diseases of the tunics remain to be considered; they will occupy an article. Another will be devoted to the anatomical portion of the volume, and then all that is susceptible of review, will have been consumed. The plates of course we cannot analyse; and here we must be contented to express our admiration. They are executed in the best style, without exaggeration, without caricature; portraying with fidelity the diseases they represent, and adding force, truth, and vivid illustration to the descriptive department of the work.

